# Understanding contact electrification at liquid–solid interfaces from surface electronic structure

**DOI:** 10.1038/s41467-021-22005-6

**Published:** 2021-03-19

**Authors:** Mingzi Sun, Qiuyang Lu, Zhong Lin Wang, Bolong Huang

**Affiliations:** 1 Department of Applied Biology and Chemical Technology, The Hong Kong Polytechnic University, Hung Hom, Kowloon, Hong Kong SAR China; 2 Beijing Institute of Nanoenergy and Nanosystems, Chinese Academy of Sciences, Beijing, China; 3 School of Materials Science and Engineering, Georgia Institute of Technology, Atlanta, GA USA

**Keywords:** Solid-state chemistry, Electronic properties and materials, Electronic properties and materials

## Abstract

The charge transfer phenomenon of contact electrification even exists in the liquid–solid interface by a tiny droplet on the solid surface. In this work, we have investigated the contact electrification mechanism at the liquid–solid interface from the electronic structures at the atomic level. The electronic structures display stronger modulations by the outmost shell charge transfer via surface electrostatic charge perturbation than the inter-bonding-orbital charge transfer at the liquid–solid interface, supporting more factors being involved in charge transfer via contact electrification. Meanwhile, we introduce the electrochemical cell model to quantify the charge transfer based on the pinning factor to linearly correlate the charge transfer and the electronic structures. The pinning factor exhibits a more direct visualization of the charge transfer at the liquid–solid interface. This work supplies critical guidance for describing, quantifying, and modulating the contact electrification induced charge transfer systems in triboelectric nanogenerators in future works.

## Abstract (web summary)

Understanding contact electrification within the liquid–solid interface is critical for further applications in energy conversion and storage devices. Here, the authors reveal liquid–solid interactions regarding the charge transfer mechanism and pinning factor from the electronic perspective.

## Introduction

To face the present global energy crisis, developing efficient energy conversion and storage (ECS) devices has been emerged as the research topic for the scientific community due to the quickly increasing demands. Among different ECS systems, the developments of the nanogenerators have presented a promising solution due to the varied modes and material selections for energy conversion^1–3^. Moreover, the superior flexibility and stretchability of nanogenerator endow the broad applications covering wearable electronics^4,5^, sensors^6,7^, energy harvesting devices^3,8^, and other self-powered electronics^9,10^. The remarkable performances of these nanogenerators depend on the piezoelectricity and triboelectricity, where the charge flow is induced by the electrical polarization and charge distribution under the applied strain or mechanical movements.

The triboelectric effect, which is also known as contact electrification (CE)-related electrostatic phenomena, is the most common situation that occurred in the ambient environment, from simple walking to even thunderstorms. The energy involved in this phenomenon has usually ignored. Moreover, based on the concept of CE, the triboelectric nanogenerator (TENG) has been invented to couple the triboelectrification and electrostatic effect for efficient energy harvesting and conversion. TENG has exhibited an advanced concept of mechanical energy harvesting, leading to a promising field of sustainable and renewable self-power electronic devices^11^. Recently, Kwak et al.^12^ applied the butylated melamine formaldehyde as a durable and highly positive friction layer for stable, high output triboelectric nanogenerators. TENG shows untapped potential in the generation of hydropower due to the flexible energy conversion modes, which maximizes the conversion energy from tiny raindrops to large water flow^13^.

The essence of the underlying mechanism of TENG is the CE effect, which results in the electricity generation simply by physical contact. Such an effect has been identified in our daily life for over 2600 years, however, it has rarely been fully utilized in the energy conversion systems^14^. Recently, the liquid–solid interaction dominated electricity generation becomes the research interest of the scientific community. The electron transfer between solids has been proved as the main source of the common triboelectrification in solid–solid systems. Wang’s group has proposed the overlapping of the electron clouds as the reason to realize the electron transfer between two materials^15^. When two solids show a distance, the electron distribution in each material follows the energy levels and is constrained in the orbitals, which cannot induce any charge transfer between solids. As the distance between material disappears, the mechanical forces lead to the formation of an overlapped electron cloud, which significantly reduces the barrier for electron transfer. Due to the electron transfer, the charged surface is formed after the separation of two solids by CE^16,17^. In comparison, the mechanism of CE on the liquid–solid interfaces are still under investigation. Owing to the fluidity and dispersibility of the liquid, the adsorption behaviors of the ions or molecules on the solid surface results in more uncertainty and complexity for the mechanism of CE.

To overcome such a challenge, lots of experiments have been carried out to explore the CE mechanism of liquid–solid interfaces from both qualitative and quantitative perspectives. The first liquid–solid contact-based TENG has been invented in 2013, which leads to the positively charged water and negatively charged polydimenthylsiloxane (PDMS) surface^18^. Such a charge originates from the ionization of the surface groups. Besides the simple contact-separation mode there are also many other liquid–solid contact types including single-electrode type^19–23^, sliding free-standing type^24–27^, pressing-releasing type^28^, and the streaming type^29–31^. Recently, Prof. Zhong Lin Wang’s group has successfully proved the existence of charge transfer between the deionized water droplet and polytetrafluoroethylene (PTFE) film^32^. By calculating the electron charge induced by the adsorption of hydroxide groups (OH^−^) groups, the excessive electrons detected in the system is attributed to the charge transfer between the liquid and solid surface.

The concept of the electric double layer (EDL) at the liquid–solid interaction becomes another promising mechanism to explain the charge transfer behaviors. Such a concept has been established by Helmholtz^29^ and further developed by Gouy and Chapman^33^. Between the formed two layers on the solid surface, the closest layer consists of the compact adsorbed ions, which is known as the stern layer. The second layer is called the diffusion layer, where the ions are highly mobile with loose binding on the surface. The flow of these charged ions induces the current output, which is detectable^34,35^. Recent experiments have confirmed that different ions in the solution lead to different current outputs^32,36^. It is also noted that as the ion concentration increases, the ion adsorption on the PTFE hinders electron transfer, which leads to a lower current output. Through the investigation between oil droplets without ions, the detected electrical current confirms the presence of charge transfer between the liquid and the solid.

To further investigate the charge transfer behaviors, we introduce the band offset to understand the charge transfer at the liquid–solid interface. Band offset, the energy difference of the valence band or conduction band, usually represents the charge transfer induced by the interfacial interactions. To precisely correlate such electronic behaviors, the “pinning factor” has been introduced to reveal the charge transfer at the semiconductor interfaces in previous works^37–40^, which is usually accompanied by chemical bonding. The deviation of the linear pinning factor indicates the potential existence of chemical bonding (chemisorption) or even chemical reactions at the interface. Based on this concept, the promising efficient liquid–solid systems can be identified for future TENG-based devices.

Herein, we have applied the density functional theory (DFT) to quantify the electron transfer in the liquid–solid systems in different oxides. Ten different oxide solids models with both pure water and solution environment have been preliminarily investigated. We also aim to correlate the charge transfer with the electronic structure changes and adsorption energies to unravel the potential affecting factors for the charge transfer. This work supplies significant references to understand the CE effect in different TENG systems, which benefit the future design of more efficient energy harvesting devices.

## Results

### Material selections

**Fig. 1:**
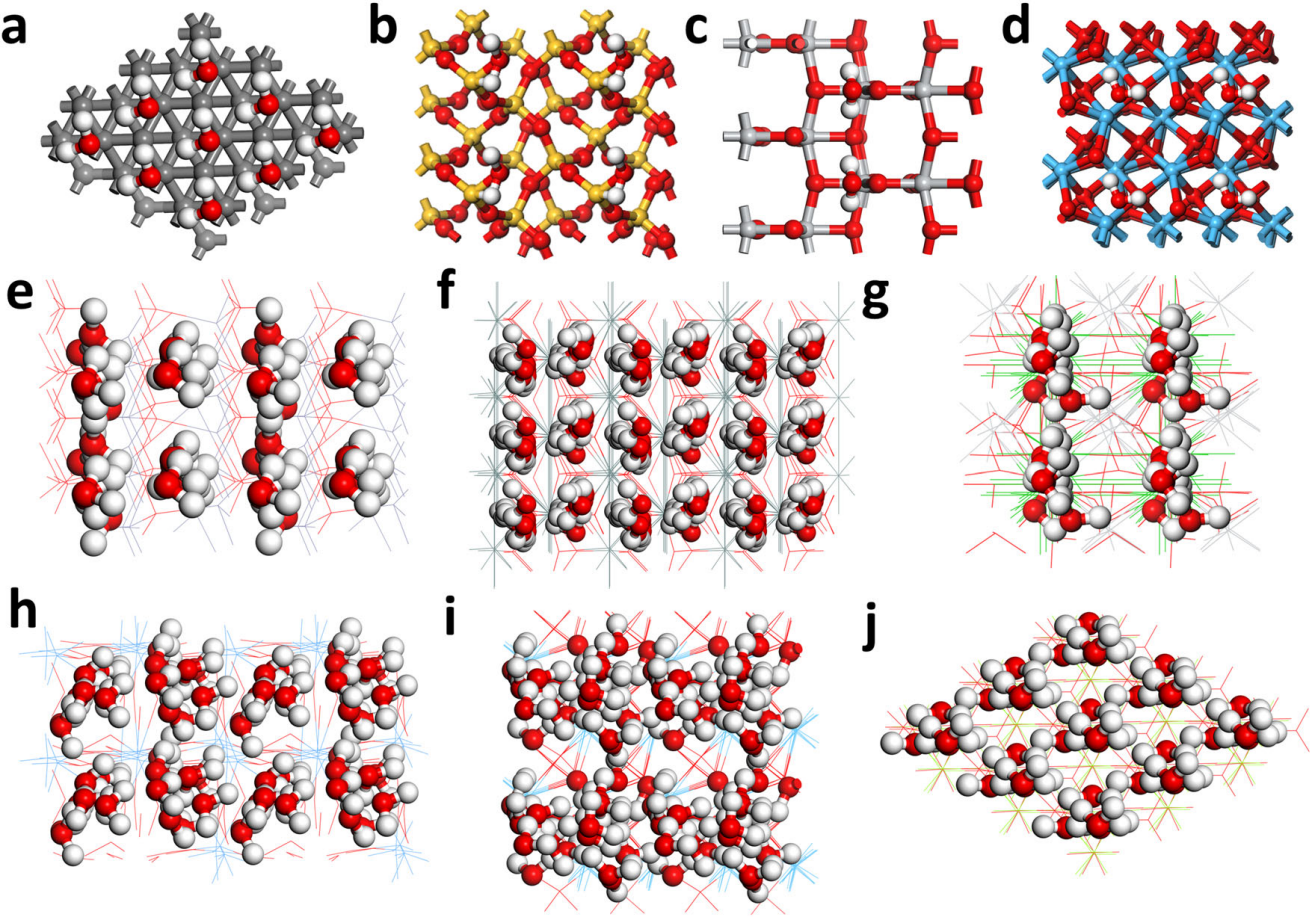
The top view of different solids after CE with water. Single-layered water on **a** Diamond, Gray balls = C, **b** SiO_2_, Yellow balls = Si, **c** TiO_2_, Silver balls = Ti, and **d** HfO_2_, Blue balls = Hf. Red balls = O and White balls = H. For the multi-layered water models, the solid materials have been displayed by lines to deliver a clear demonstrations. Multi-layered water on **e** ZnO, Dark silver line = Zn, **f** SnO_2_, Grey line = Sn, **g** MgO, Light green line = Mg, **h** HfO_2_, Blue line = Hf, **i** Ta_2_O_5_ Light blue line = Ta, and **j** BaTiO_3_, Green line = Ba. Red balls = O and White balls = H.

In previous works by Prof. Zhong Lin Wang and his team, the CE of the liquid–solid interface, as well as the potential mechanism, have been studied on the PTFE films^32,41,42^. In these works, they have proposed different charge transfer mechanisms based on different solutions and ion concentrations. Later, the charge transfer has been quantified in several transition metal oxides after contacting the liquid water^41^. Therefore, considering the recent progress by experiments, we decided to investigate the CE at liquid–solid interfaces on the oxide materials regarding the electronic structures from atomic view (Supplementary Figs. 1 and 2). We have considered single-layered and multi-layered water layers on different solid surfaces. For the single-layered water, we choose the diamond, SiO_2_, TiO_2,_ and HfO_2_ with different dielectric functions (Fig. 1a–d and Supplementary Fig. 3a–d). For the multi-layered water, we have chosen more oxides including ZnO, SnO_2_, MgO, HfO_2_, Ta_2_O_5,_ and BaTiO_3_ (Fig. 1e–j and Supplementary Fig. 3e–j). It is noted that the lattice structures of most solids are preserved. However, as the number of water layer increases, the interactions at liquid–solid interfaces become much stronger. Such strong interactions result in the interruption of the long-range order of water molecules. Meanwhile, the lattice structures of different solids are also distorted, which is accompanied by the adsorption of water molecules to passivate the surface dangling bonds.

### Liquid–solid interface interactions

Then, we further investigate the electronic structure of each liquid–solid interface. Although the charge transfer between the solid and liquid surfaces is relatively small, the total density of states (TDOS) of the liquid–solid surfaces is able to reflect the charge density variation, which is sensitive to the interactions and environments (Supplementary Fig. 4a–c). Therefore, we firstly apply the TDOS approach to evaluate the charge transfer behaviors. For the pure insulators, such as carbon, we can notice that, moving from the bulk to the surface layer, the electron density becomes evidently larger at the Fermi level (*E*_F_). After the introduction of water molecules, no band offset has been noticed. Instead, a slight decrease in the states pinned at *E*_F_ is noticed. When the Na ions are introduced to the interlayer space of the water layers, the passivation on the carbon surface becomes more evident, where the pinned states only appear at the bulk. In comparison, the passivation of the dielectric insulator SiO_2_ by the single-layered water molecules also exists (Supplementary Fig. 4d–f). The similar pinned states disappear when the single-layered water interacts with the surface. With the presence of Na ions, the liquid–solid surface band offset occurs, in which the surface layer downshifts nearly 4 eV. From the surface to the bulk, such downshifting becomes weaker, supporting the formation of an electronic double layer (EDL) due to the charge transfer from contact between the solution and the solid surface. Then, we further look into the transition metal oxides TiO_2_ (Supplementary Fig. 4g–i). Interestingly, the TDOS becomes more sensitive, leading to a band offset of 1 eV after contact with the water molecules. Similarly, the introduction of Na ions causes the gradual change of the TDOS from the surface to the bulk. Compared to the dielectric oxide, the band offset is much smaller. Then, for the high-k oxide HfO_2_, the influences on the electronic structures of layers are very weak, where the band positions barely change (Supplementary Fig. 4j–l). Even with the introduction of Na ions with a positive charge, the charge transfer dominated band offset is observed. Through the quantitative band offset of the TDOSs, the evidently different changes induced by contact with water and solutions are unravelled.

**Fig. 2:**
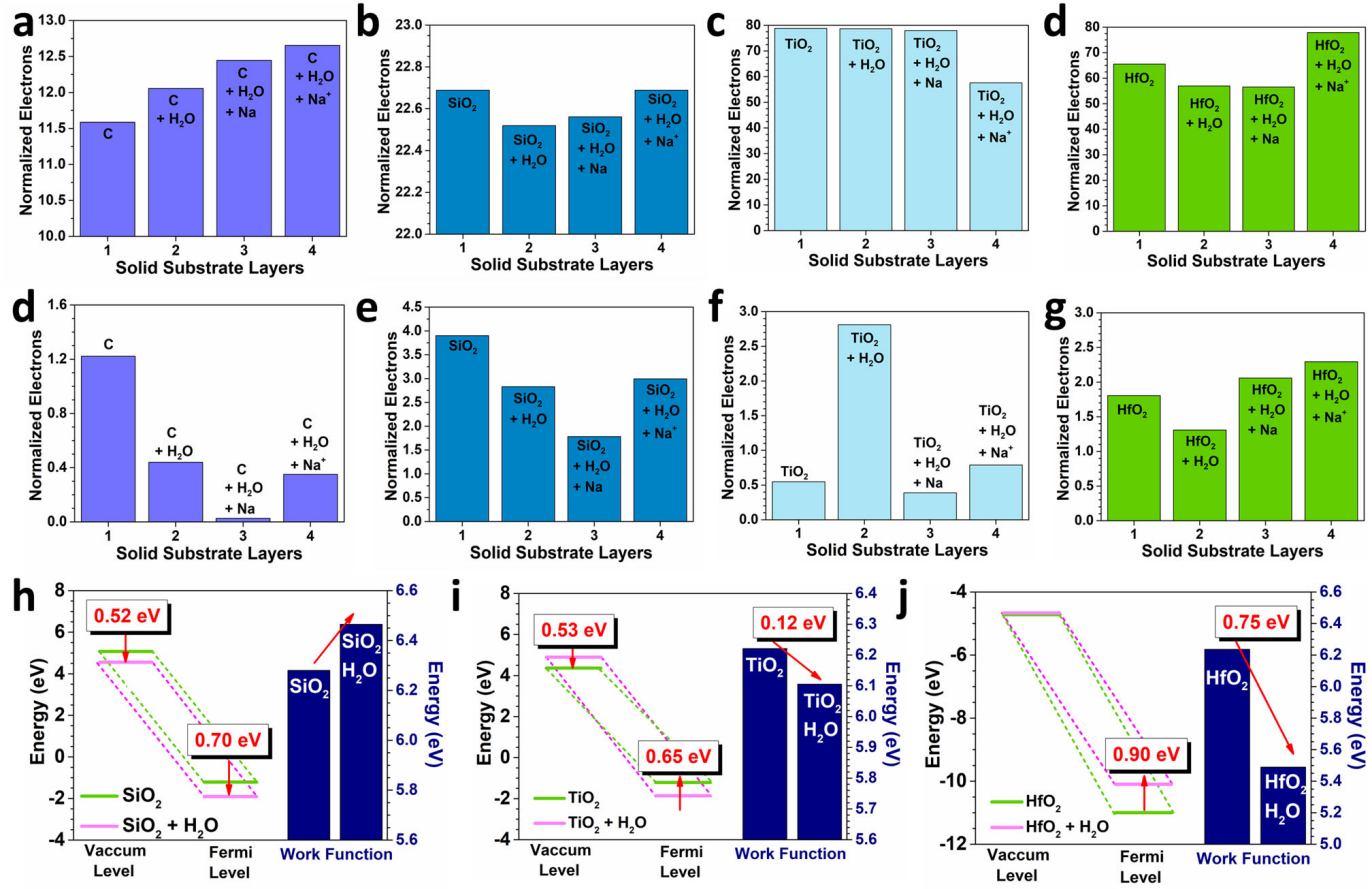
The normalized electron numbers of the valence band for different solids in contact with water and solution. **a** Diamond carbon, **b** SiO_2_, **c** TiO_2_, and **d** HfO_2_. The normalized electron numbers near the Fermi level for different solids in contact with water and solution. **d** Diamond carbon, **e** SiO_2_, **f** TiO_2_, and **g** HfO_2_. **h** The comparison of vacuum level, Fermi level change, and work functions after contacting with water. **h** SiO_2_, **i** TiO_2_, and **j** HfO_2_.

From the TDOS band offset, we notice the interactions between solid and liquid as well as the corresponding charge transfer. To further understand the charge transfer induced by the contact, we apply two different calculations to reveal the electrons change and transfer directions in the valence band (VB) of these solid with different electric constants. The first one we consider the electron number changes in the whole VB of the solid. On the other hand, we only evaluate the electron number of changes in a small range near the *E*_F_, which represents the most possible and active electron transfer region. For the whole VB, we notice that the diamond surface shows a continuously increasing trend of the electron numbers as contacting with more and more ions (Fig. 2a). For the SiO_2_ surface, the contact with water molecules causes the decreases of the electron numbers while the introduction of Na ions increases the electron number (Fig. 2b). This might indicate a converse electron transfer direction between SiO_2_/water and SiO_2_/Na. Such a phenomenon counteracts the overall electron changes, which is consistent with the nearly unchanged over electron number for SiO_2_ and SiO_2_ + H_2_O + Na^+^. Converse to the pure carbon, the TiO_2_ shows a continuous downhill trend for the electrons (Fig. 2c). This indicates an electron depletion trend for the solid surface, especially when the surface is interacting with the positively charged surface. The HfO_2_ surface displays a similar trend with the SiO_2_ surface, which also supports the overall different electron transfer direction (Fig. 2d). However, when we only consider the electron change near *E*_F_ (*E*_V_−1.0 eV to *E*_V_ + 1.0 eV, *E*_V_ = 0 eV), we notice a different variation change. For the carbon surface, a decreasing trend of the electron numbers is noted, which is converse to the overall change in the VB (Fig. 2e). A similar converse trend is also noted for the SiO_2_ surface (Fig. 2f). Notably, the contact of TiO_2_/water induces great increases in the electron density (Fig. 2g). The sudden decrease of the electrons originates from the introduction of Na ions. The electron changes of HfO_2_ exhibit a similar trend in both small and large energy range, which confirms a consistent electron transfer behavior (Fig. 2h). As further evidence of the formation of EDL on the liquid–solid surface, we compare the detailed electronic structures of the oxides (Fig. 2i, j). By contacting with the single-layered H_2_O, the changes of both the vacuum level and Fermi level of the solid surface display distinct trends in different oxides, indicating different modulations for electron transfer. The work functions also show completely different behaviors. For SiO_2_, the increase of work function demonstrates the electron trapping near the solid surface. For TiO_2_, a slight decrease in the work function leads to easier electron transfer from the VB on the near-surface. For HfO_2_, an evident alleviation of the work function demonstrates the electrons are easily transferred from the solid surface to water molecules. However, the increase of the Fermi level also indicates the enlarged barrier for electrons. The balance of these two effects leads to the weaker electron transfer for both TiO_2_ and HfO_2_.

**Fig. 3:**
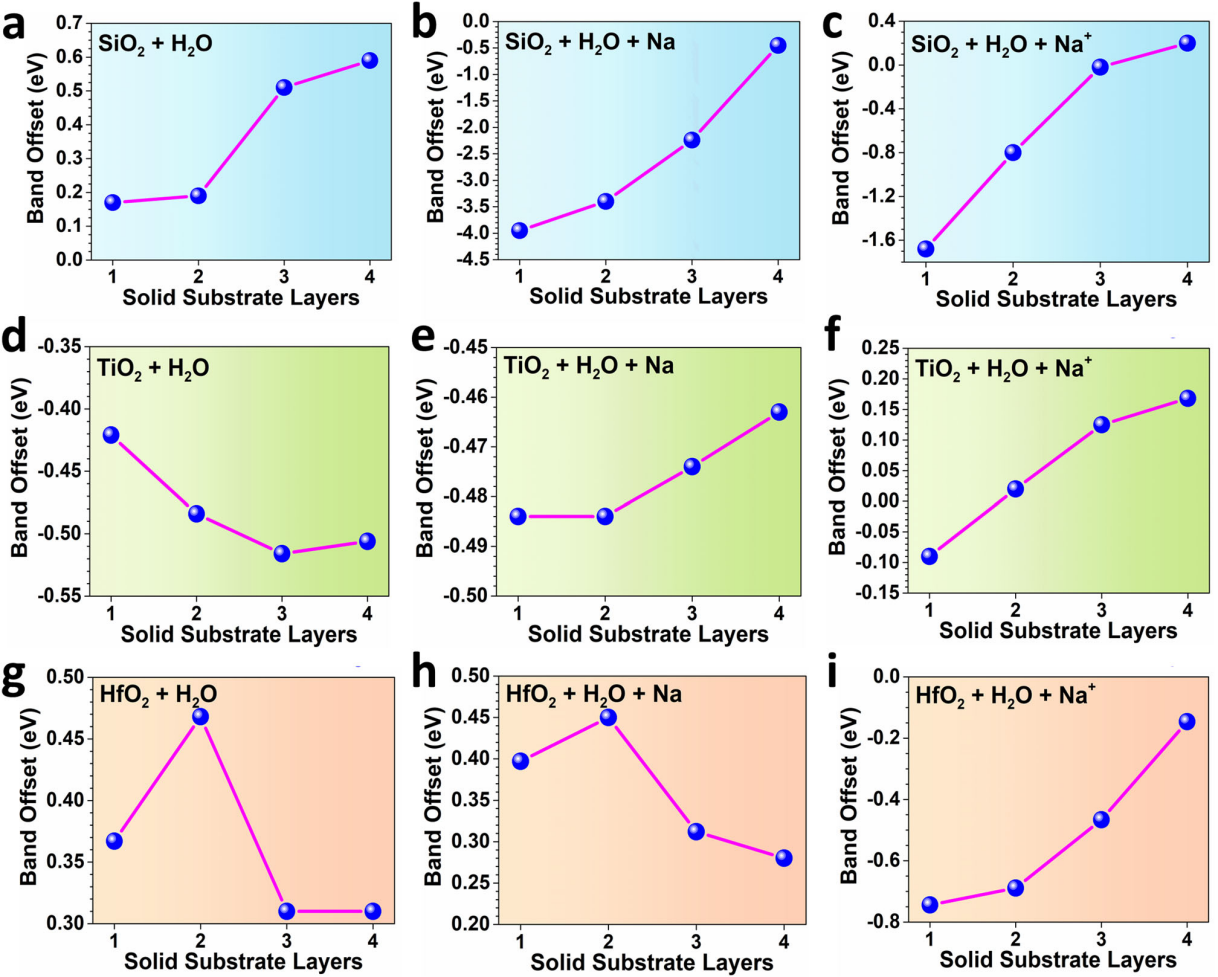
The band offset variations for different layers of liquid–solid systems. **a** SiO_2_ + H_2_O. **b** SiO_2_ + H_2_O + Na. **c** SiO_2_ + H_2_O + Na^+^. **d** TiO_2_ + H_2_O. **e** TiO_2_ + H_2_O + Na. **f** TiO_2_ + H_2_O + Na^+^. **g** HfO_2_ + H_2_O. **h** HfO_2_ + H_2_O + Na. **i** HfO2 + H_2_O + Na^+^.

Then, the detailed band offset has been quantitively compared from the surface layer to the bulk layer. For SiO_2_, TiO_2,_ and HfO_2_, three oxides show different band offset levels on the contact with water. Based on the band offset scale, SiO_2_ shows the highest sensitivity to the local environment (Fig. 3a–c). For the contact with pure water molecules, the solid shows nearly the same band offset from the surface to the bulk. As the Na ions are introduced, the drastic downshifting of 4 eV to the band offset is demonstrated. Even for the bulk structure, the TDOS also shift towards the lower position of 1.0 eV. For the TiO_2_, we notice that the Na ions lead to a converse trend of the band offset (Fig. 3d–f). However, compared to SiO_2_, such a band offset range is much smaller, indicating the relatively inert properties of the water and Na ions. Meanwhile, although the overall trend is similar, the positively charged Na ions shift the TDOS towards to *E*_F_. In addition, the HfO_2_ is inert to both the water molecules and solution, where the band offsets are remained highly similar (Fig. 3g–i). However, as the charged ions are induced, the surface layer downshifts ~1.2 eV, which shows a similar uphill trend of the band offset from the surface to the bulk. Based on these results, the charged Na ions show stronger impacts on the band offset than the pure molecules, which support the contribution of the ion concentrations to the charge transfer in the contact electricity of liquid–solid interface.

To further simulate the practical environment of EDL, we further increase the water layer and investigate the impacts on more different oxides. Through the PDOS, the electronic structure change has been demonstrated. The pristine SnO_2_ demonstrates a barely changed electronic structure from the surface to the bulk (Supplementary Fig. 5a–c). The introduction of the thick water layer leads to the slight downshift of the VB. Notably, the introduction of Na ions significantly increases the overall electronic density, where the subtle band offset has been annihilated. ZnO also exhibits limited change after contacting with the multi-layered water or solution (Supplementary Fig. 5d–f). Except for the surface layer, no evident band offset is noted from the sublayer to the bulk, indicating the limited charge transfer. Meanwhile, MgO demonstrates a highly flexible electronic structure, which is strongly affected by surface contact with water or ionic solution (Supplementary Fig. 5g–i). Although the electronic structure has been varied significantly, the band offset is absent. These results support less controllable charge transfer behaviors as the intrinsic nature of MgO, which is not directed by the formation of EDL. The evident electronic densities changes are noted in both MgO and SnO_2_ solid surface after the introduction of Na. Such evident variations of electron density are attributed to the stronger electron transfer by the p-p couplings between solid and Na ions. In comparison, the p-d couplings on ZnO surfaces are limited even after the introduction of Na ions, which cannot induce the obvious increases in electron density.

For Ta_2_O_5_, contacting with water does not induce any evident band offset or charge density variation (Supplementary Fig. 6a–c). However, when meeting with ionic solution, the 1.5 eV band offset occurs with the enhanced charge density. A gap state is even noticed for the bulk layer of Ta_2_O_5_. For the multicomponent oxides BaTiO_3_, we notice the evident charge density increases after contacting with water and solution (Supplementary Fig. 6d–f). BaTiO_3_ demonstrates an overall band offset through the downshifting of 0.75 eV. Compared to the solution, the interaction with water mainly contributes to the band offset since the further introduction of ions only affects the charge density rather than modulating the band offset. In the initial models, the HfO_2_ with one layer of water displays very weak sensitivity to the interaction with both water and the charge transfer (Supplementary Fig. 6g–i). As the thickness of the isolution lay/solution layer increases, the charge transfer within the liquid–solid interface becomes much more evident. For pure water molecules, the band offset in HfO_2_ is barely noticed with limited changes on charge density. When the ion concentration becomes higher, the presence of a large band offset ~3.0 eV is noted. However, such a band offset is similar for all the layers, which shows no degradation phenomenon of conventional EDL.

**Fig. 4:**
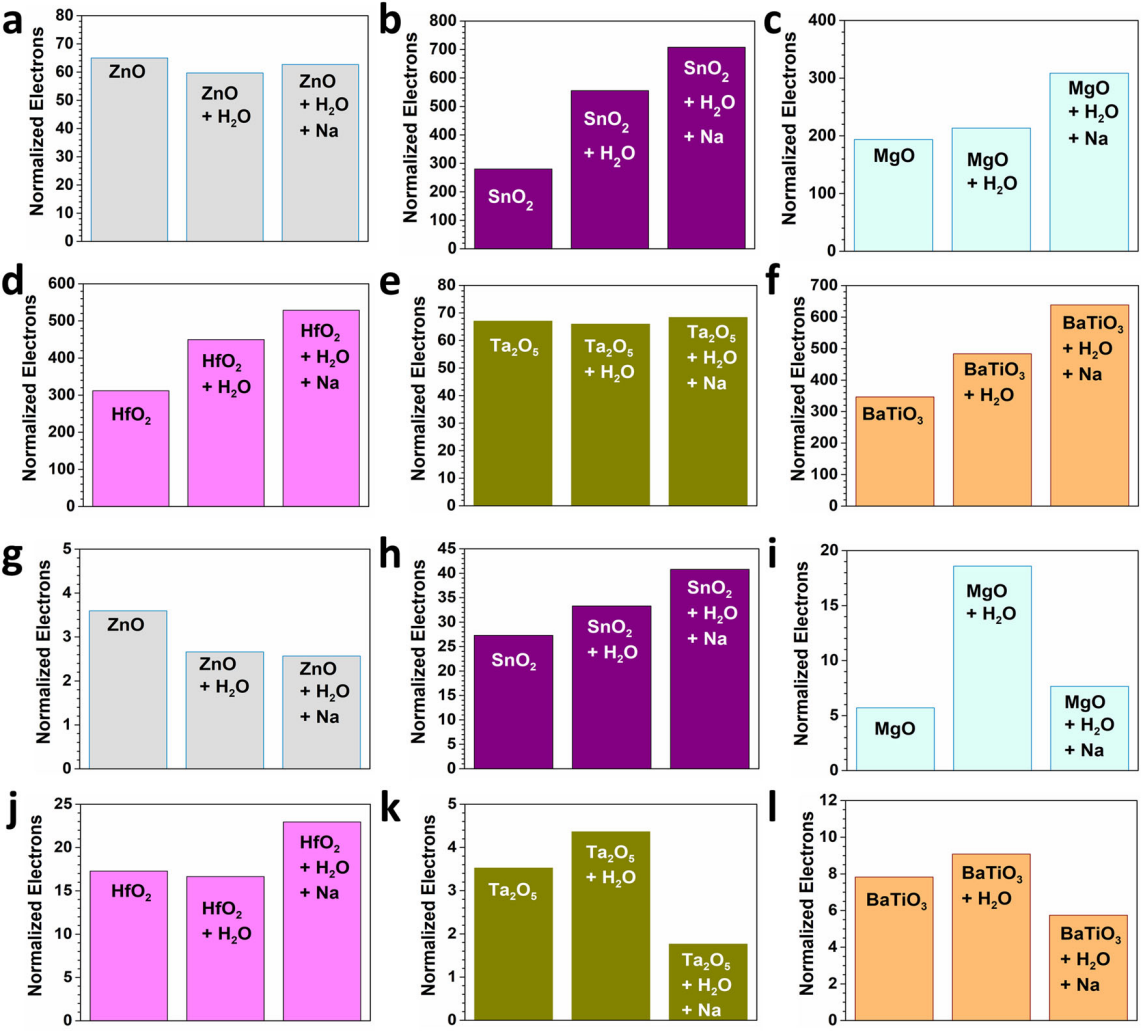
The normalized electron numbers of the valence band for different solids in contact with water and solution. **a** ZnO, **b** SnO_2_, **c** MgO, **d** HfO_2_, **e** Ta_2_O_5_, and **f** BaTiO_3_. The normalized electron numbers near the Fermi level for different solids in contact with water and solution. **g** ZnO, **h** SnO_2_, **i** MgO, **j** HfO_2_, **k** Ta_2_O_5_, and **l** BaTiO_3_.

Through a similar method, the electron density has been compared regarding the whole VB and the small range near the *E*_F_ (*E*_V_ − 1.0 eV to *E*_V_ + 1.0 eV). SnO_2_ demonstrates the continuous increasing trend of the electron ions, indicating the strong charge transfer to the surface by the formation of EDL (Fig. 4a). On the contrary, for ZnO, the introductions of the water molecules and Na ions induce the opposite effect to the variation trend of electron number in the system (Fig. 4b). The introduction of water reduces the electrons while the Na ions slightly increase the electron number. For MgO, both water and Na on the solid surface have increased the electron numbers of VB (Fig. 4c). The contact with water molecules and solution barely affects the change of electron numbers in the whole VB (Fig. 4d). Combined with the TDOS results, such liquid–solid interaction only induces the band offset without modulating the electron numbers. For both BaTiO_3_ and HfO_2_, we notice the increasing trend for the electron numbers induced by both water molecules (Fig. 4e, f). Contacting with water molecules only increases the electron number change while the Na ions enable the band offset, indicating the different influences by the contact. However, the electron number change near *E*_F_ demonstrates a different trend. The electron occupations in SnO_2_ still demonstrates a similar variation trend as the whole VB, which confirms the absence of band offset (Fig. 4g). In comparison, ZnO displays the downhill trend for contacting with water molecules and Na ions (Fig. 4h). This interprets that water molecules dominate the charge transfer instead of the Na ions. Interestingly, we notice the complete opposite trend of electron transfer for MgO in the whole VB and a small range near the *E*_F_ (Fig. 4i). Such a contrast further confirms the less controllability of the charge transfer, which is consistent with the TDOS results. Although Ta_2_O_5_ shows the nearly unchanged electron numbers in the whole VB even after the liquid–solid interaction, the electroactive electrons near *E*_F_ are affected by the contact (Fig. 4j). The converse contribution to the electron number results in the subtle change of the electron numbers. The increases of electrons in BaTiO_3_ verified the contribution of water (Fig. 4k). Meanwhile, the sudden decreases of the electrons by the Na introduction is ascribed to the band offset. The different influences of water and solution are revealed. It is noted that the thicker water layer does not change the electron number much in HfO_2_ (Fig. 4l). Owing to the overall band offset from surface to bulk layer, the Na introduction still increases the electrons. Compared to the single-layered water model, the multi-layered model is more regulated, which will be applied to the further quantification of the charge transfer.

### Electronic structure comparison

To directly visualize the electronic structure change induced by the liquid–solid interaction, we first compare the electronic change of surface water on different solids after CE. For the single-layered water, it is noted that the p-*π* electronic distribution of water has been significantly modified and redistributed. On the diamond surface, the *s,p* orbitals of water molecules have been suppressed to a lower position. On both TiO_2_ and SiO_2_, water molecules have shown more concentrated electron density near the *E*_F_. The single-layered water on HfO_2_ also shows a similar electronic structure but in a deeper position (Supplementary Fig. 7a). With more water molecules are accumulated near the solid surface, the electronic modulations by the charge transfer within the liquid–solid interface are more evident. The multi-layered stacking of water molecules shows the continuous electron density that crosses the *E*_F_. However, after contact with the solid surface, the modulations of the electronic distribution still exist near the solids while non-contact water molecules still preserve the p-*π* bonding. Notably, the ZnO, SnO_2_, MgO, HfO_2,_ and Ta_2_O_5_ display similar modulations of the *s,p* orbitals with an evident gap between the valence band and conduction band. In comparison, BaTiO_3_ still maintains the high electron density near the *E*_F_, indicating a more complicated modulation (Supplementary Fig. 7b). The stronger surface orbital coupling leads to the chemisorption of water molecules and the deviation from the pinning factor. Moreover, the adsorption of the water molecules not only passivate the surface dangling bond but also induces the shielding effect as proposed by Prof. Zhong Lin Wang^32^. Therefore, for both single-layered and multi-layered water on the solids, the evident PDOS changes confirm the existence of charge transfer.

On the other side, we also compare the PDOS of different solid surfaces in Supplementary Fig. 8. For single-layered water on both diamond and HfO_2_, we notice a highly similar PDOS pattern, especially for HfO_2_. The overall electronic structures have remained the same with subtle electron number variations. On the other side, we notice the opposite shift directions on surfaces of SiO_2_ and TiO_2_. Although the electron densities have not been significantly changed, the band shifting demonstrates the opposite electron transfer directions on these two surfaces (Supplementary Fig. 8a–d). As the water layer becomes thicker, the changes in the PDOS become more evident and complicated. For the ZnO, although the band positions are maintained, the electron density shows an overall increasing trend (Supplementary Fig. 8e). Both SnO_2_ and MgO surfaces exhibit significant modulations by the liquid surface. For SnO_2_, the broad *s,p* orbitals become sharp peaks near the *E*_F_. (Supplementary Fig. 8f, g). For both HfO_2_ and Ta_2_O_5_, the electron densities show similar band positions and electron densities (Supplementary Fig. 8h, i). From the detailed quantification of electron numbers, the electron numbers of both surfaces slightly increase as shown in Fig. 3. A similar result is also identified in BaTiO_3_ with an upshifting trend of the PDOS (Supplementary Fig. 8j). These results confirm different types of electronic modulations induced at the liquid–solid interface.

In addition, to further visualize the electron transfer trend, we have also supplied the electron density difference (EDD) results of all the liquid–solid surfaces in Supplementary Fig. 9. For the single-layered water (Supplementary Fig. 9a–e), we notice the limited influence on the p-*π* electron cloud of water. Meanwhile, the electron cloud of the solid surface has also been slightly modified. For the multi-layered stacking of water on different surfaces (Supplementary Fig. 9f–l), distinct electronic behaviors are noted. For ZnO, even close to the liquid layer, the electron density is not evidently affected when compared to the bulk. In comparison, the electron densities of surface layers for MgO and SnO_2_ are perturbed by the surface water layers. The relatively strong interactions at the liquid–solid interface also lead to the electronic redistribution of surface water even to those non-contacting waters with distances. For HfO_2_, even with an increased water layer, the electronic distribution of water is strongly affected while the solid surface shows a limited change. For Ta_2_O_5_ and BaTiO_3_, the electronic distributions of solid surfaces are both slightly weakened. However, the charge transfer effect shows a stronger perturbation of the well-ordered water stacking in the Ta_2_O_5_ than BaTiO_3_. The electron transfer trends are supportive of the quantification results.

### Electrochemical cell model

From the conventional EDL mechanism proposed by Gouy and Chapman^33^, the thickness *L* of the diffusion layer has been described as Eq. (1).

1 $$L^{{\mathrm{ - 1}}} = \left( {\frac{{\varepsilon _0\varepsilon {{kT}}}}{{2n_i^0c^2e^2}}} \right)^{{\mathrm{1/2}}}$$

In the above expressions, the $\varepsilon _0$ and $\varepsilon$ represent the permittivity of vacuum, *ε* is the relative permittivity of the material. *k* is the Boltzmann constant and *T* is the absolute temperature.$n_i^0$ indicates the ions concentration and the *c* represents the charge on the ions. *e* is the electron charge.

However, based on such an equation, the detection of the EDL by CE in the liquid–solid interface becomes challenging due to the coupling of the adsorption effect and shielding effect in the CE^32,41^. Therefore, we decide to propose a different approach to understand the charge transfer, where the liquid–solid interface has been considered as a cell. In such a cell, the charge transfer between electrodes is induced by the potential difference, which is shown as the work function variations. The mechanism is expressed as below.

2 $$E_{{\mathrm{EDL}}} = \varphi _i = {{\int_{0}}^{x}} {\varphi _i^0{\mathrm{exp}}\left( { - \kappa {\it{L}}} \right) = z \,\times\, {\mathrm{charge}}}$$

In Eq. (2), $\varphi _i$ represents the integration of the potential gradients and *z* represents the electron transfer numbers. *L* is the ion diffusion layer thickness.

By the Gibbs free energy definition in physical chemistry and the theoretical calculations, we have the following equations.

3 $${\Delta}{{G}} = {\Delta}{{H}} - {{T}}{\Delta}{{S}} + {\Delta}{\mathrm{ZPE}} = - z{\mathrm{F}}E_{{\mathrm{cell}}}$$

4 $$\left| z \right| = \left| {\frac{{{\Delta}{{G}}}}{{ - {\mathrm{F}}E_{{\mathrm{cell}}}}}} \right|$$

Since we have considered the liquid–solid interface as a cell, *E*_cell_ is obtained by the difference of the work function induced by the contact with pure water. The charge is estimated regardless of the direction.

In this work, the ${\Delta}{{G}}$ is demonstrated by the average adsorption energies of water molecules in the liquid–solid interface. By further dividing the Avogadro’s number, we are able to further quantify the charge transfer in different liquid–solid interfaces. The summarized charge transfer and other parameters are listed in Supplementary Table 1, which shows a similar level with the experiment characterizations^32^.

By utilizing the pinning factor, the charge transfer barriers and the chemical bonding trends have been directly visualized. At the initial investigation, it is believed that such a critical factor relies on electronic conductivity. Later, Mönch has proved that the dielectric function is strongly linked with this factor in different interfaces^37–40^. Therefore, the identification of the significant pinning factor *P* enables the classification of charge transfer for future TENG-based devices. The pinning factor *P* usually varies between 0 (for a strongly pinned interface, i.e., Bardeen limit) and 1 (for no pinning interface, i.e., Schottky limit)^40,43^. The Taylor expansion is shown in Eq. (5) below, where the first term is the dominant contribution to *P*.

5 $$P = \frac{1}{{1 + 0.1\left( {\varepsilon - 1} \right)^2}} + \frac{{ - \frac{{0.2\left( {\varepsilon - 1} \right)}}{{\left( {1 + 0.1\left( {\varepsilon - 1} \right)^2} \right)^2}}}}{{1!}}{\varepsilon} + \ldots \cdots$$

**Fig. 5:**
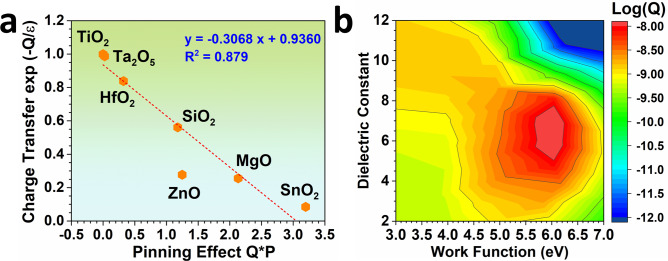
The correlations between charge transfer at the liquid–solid interface and other parameters including pinning factor, work function and dielectric constant. **a** The linear correlation between charge transfer and dielectric function that is based on the introduction of the pinning factor. *Q* represents the charge transfer, *ε* represents the dielectric constant of the materials. *P* represents the pinning factor. **b** The correlation between work function, calculated dielectric constant, and charge transfer (*Q*).

Since the dielectric function of the lid surfaces indeed plays an important role in the EDL, we also combine it into the correlation, where a linear relationship has been demonstrated (Fig. 5a). It is noted that most of the models locate on the linear line, which shows a fitting coefficient of 0.996. Such a linear fitting supports that the charge transfer is highly correlated with the dielectric function and adsorption energies. The deviation of ZnO and SnO_2_ from the linear correlation is attributed to the ultra-low sensitivity to the CE contact in the liquid–solid system, which also significantly affects the other terms in Eq. (5). This is attributed to the residue charges originated from the dominant chemisorption. The correlation between the charge transfer and the work function is also demonstrated in Fig. 5b. It is noted that a volcano trend existed, in which the higher work function does not directly lead to the large barrier for charge transfer. At the higher work function, the dielectric function shows an important influence on the charge transfer. The maximum charge transfer shows a similar level as previous experimental characterizations^32,41^.

Furthermore, if we consider the entropy in Eq. (3), we are able to classify the entropy into two different types: the cell potential induced elastic entropy and the contact induced elastic entropy, which contributes to the potential and charge, respectively. The further derivations of these two types of entropy are shown as Eqs. (6) and (7).

6 $${\mathrm{Elastic}}\,{\mathrm{entropy}}\,\left( {{\mathrm{potenitial}}} \right):\left[ { - T\delta S_{{\mathrm{elastic}}}} \right] = - {\mathrm{F}}\left( {E_{{\mathrm{cell}}}\delta z + z\delta E_{{\mathrm{cell}}}} \right)$$

7 $${\mathrm{Elastic}}\,{\mathrm{entropy}}\left( {{\mathrm{contact}}} \right)\,:\,S = \frac{{qQN_A}}{{8\varepsilon _s\left( {T - T_i} \right)}}x^2$$

In Eq. (7), $\varepsilon _s$ indicates the dielectric function, *x* represents the perturbation displacement, and *Q* is the perturbation charge^44^.

In the current work, we applied the work function change induced by the CE to represent the electrode potential difference in the cell for the estimation of the charge. However, since other potential parameters also are influenced by the CE such as the vacuum level and Fermi level, we have considered a more complicated correlation between the charge and the dielectric function by applying the different potential differences in Eq. (4) to estimate charge transfer induced by the contact with water.

**Fig. 6:**
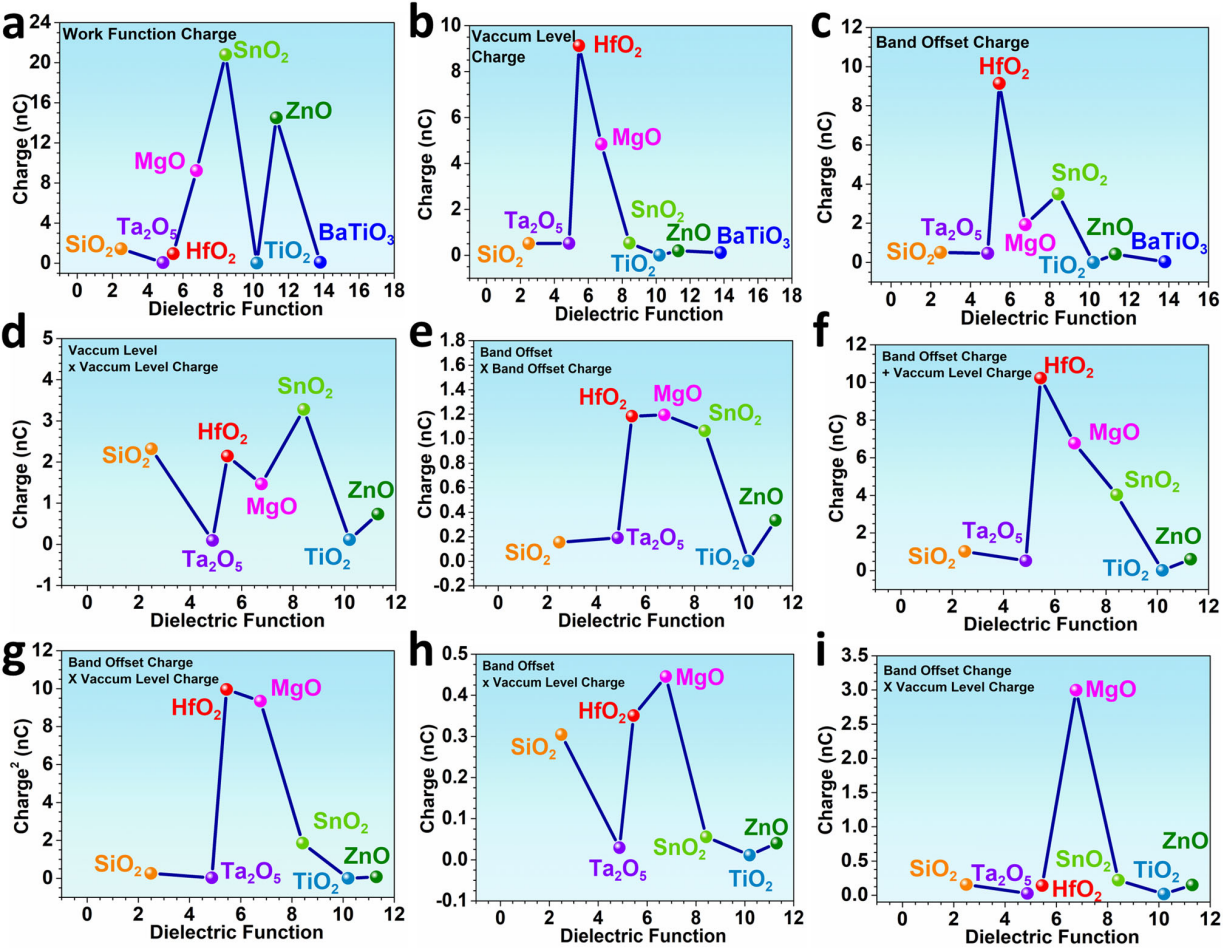
The correlations between the dielectric functions and charge transfer which is calculated by different potential parameters. **a** The work function. **b** The vacuum level. **c** The band offset. **d** The vacuum level × the vacuum level. **e** The band offset × the band offset. **f** The band offset × the vacuum level. **g** The band offset calculated charge × the vacuum level calculated charge. **h** The band offset × the vacuum level calculated charge. **i** The band offset variation × the vacuum level charge.

To correlate the direct correlation between the charge transfer and dielectric function, we have proposed nine different situations. For the calculated charge, we have applied three different cell potentials derived from the work function, vacuum level, and band offset. However, between these calculated charge results and the dielectric function of the solid surface, there are no obvious regulations, supporting more complicated parameter is needed for the charge transfer evaluation (Fig. 6a–c). When we apply the charge calculated by more complicated potential combinations, it is noted that the charge has been varied significantly, indicating that the applied potential is the key factor to influence the calculated charges (Fig. 6d–f). Even we further introduce more coefficients to the calculated charge, no evident correlation is identified between the charges and dielectric function. Among different conditions of the calculated charge transfer, we noticed that HfO_2_ and MgO show a relatively high charge value under different correlation situations, supporting the higher potential to be applied in such CE systems. More importantly, the lack of direct correlation between the charge transfer and the dielectric function indicates the further investigation of the charge quantification. Especially when the simple oxides become multicomponent oxide, more complicated factors impose an impact on the pinning factor (Fig. 6g–i).

### Derivation of charge transfer mechanism

Besides the dielectric function, the geometry parameters of the liquid–solid interface are also crucial for the charge transfer. In experiments, the contact angle of the liquid droplet also affects the charge transfer, which is related to the contact area and angle^41^. However, two types of situations should be considered. When the droplet is ultra-small and the surface tension dominates the contact, the contact area *A* is expressed as below.

8 $$A = {\uppi}\left( {\frac{{3V}}{{\pi {\mathrm{tan}}^2\frac{\theta }{2}\left( {\frac{3}{{{\mathrm{sin}}\theta }}{\mathrm{ - tan}}\frac{\theta }{2}} \right)}}} \right)^{\frac{2}{3}}$$

In the abovementioned expression, *V* represents the volume of the droplet and *θ* indicates the contact angle between the droplet and the solid surface. On the other hand, when the volume of the droplet becomes larger or under pressure, the gravity results in the flattening droplet and determines the contact area of the droplet, which leads to a distinct derivation of the contact area *A* as shown in Eq. (9).

9 $${{A}} = \frac{{\sqrt {\frac{{\rho {{g}}}}{\gamma }} }}{{2{\mathrm{sin}}\frac{\theta }{2}}}\,{{V}}$$

In this equation, *ρ* indicates the liquid density, *g* is the acceleration of gravity, and the weak surface tension is expressed by *γ*. When the contact angle *θ* is fixed, the increase of liquid only enlarges the contact area *A* instead of the thickness of the liquid layer.

By considering the contact area, we derive the charge transfer from Eqs. (2) and (3).

10 $$E_{{\mathrm{EDL}}} = \varphi _i = {{\int_{0}}^{x}} {\varphi _i^0{\mathrm{exp}}\left( {{\mathrm{ - }}\kappa {{L}}} \right)} = \frac{{{\Delta}{{G}}}}{{z \times {\mathrm{e}}}}$$

11 $${\mathrm{ln}}\,\varphi _i^0 - \kappa {{L}} = \frac{{ - z {\mathrm{F}}E_{{\mathrm{EDL}}}}}{{z \times {\mathrm{e}}}}$$

By introducing Eq. (1) in Eq. (11), we have the following expression for *c* = ±1 charge on each ion such as OH^−^ and H^+^.

12 $$- \left( {\frac{{2n_i^0e^2}}{{\varepsilon _0\varepsilon {{kT}}}}} \right)^{\frac{1}{2}}{{L}} = {\mathrm{ln}}\frac{{ - {\mathrm{F}}E_{{\mathrm{EDL}}}}}{e} - {\mathrm{ln}}\varphi _i^0 = {\mathrm{ln}}\frac{{ - {\mathrm{F}}E_{{\mathrm{EDL}}}}}{{{\mathrm{e}} \times \varphi _i^0}}$$

For a certain liquid–solid interface, the *E*_EDL_ and $\varphi _i^0$ should be constant and we apply constant D to represent the right constant term. Thus, the diffusion length of the work is demonstrated in Eq. (13).

13 $${{L}} = - {\mathrm{D}} \times \sqrt {\frac{{\varepsilon _{\it{0}}\varepsilon kT}}{{2n_i^{\it{0}}}}} \times \frac{1}{e}$$

In previous work, the charge transfer by the contact has been calculated by Eq. (14) ^32^.

14 $$Q_s = {\mathrm{2e}}N_{\mathrm{A}}{{cL}}A$$

Then, we introduce both the diffusion layer thickness and contact area into Eq. (14) and determine the optimized description of charge transfer expression as Eq. (15).

15 $${{Q}} = - {\mathrm{D}} \times \sqrt {\frac{{2\varepsilon _0\varepsilon kT}}{{n_i^0}}} \times \pi \left( {\frac{{3V}}{{\pi {\mathrm{tan}}^{\mathrm{2}}\frac{\theta }{2}\left( {\frac{3}{{\sin \theta }} - \tan \frac{\theta }{2}} \right)}}} \right)^{2/3} \times N_{\mathrm{A}}c$$

Therefore, the actual charge transfer within the liquid–solid system is much more complicated than previous works, which involves the contact angle, dielectric function, and temperature, ion concentration, etc. Based on our proposed electrochemical cell model, the evaluation of the charge transfer has been preliminarily investigated through different approaches, which demonstrates the complicated mechanism for the CE within liquid–solid systems. This work indicates the diverse factors involves in the liquid–solid interface, which explains why the CE within the liquid–solid interface cannot be well fitted by conventional EDL or charge transfer models. Further in-depth investigations are still needed to determine the key factors affecting the charge transfer in CE to promote the performances of TENG in an extra wider range of applications.

## Discussions

In this work, we have investigated the electron charge transfers within the different liquid–solid systems, which are dominated by the CE effect. Compared to the conventional EDL models by Gouy and Chapman, the proposed TDOS approach in our work shows more complicated factors and varied trends of the charge transfer in the liquid–solid interface. Based on our proposed electrochemical cell model regarding the work functions and adsorption energies, a linear correlation has been identified with the introduction of the pinning factor, under idealized conditions. The pinning factor reveals that strong chemisorption of water molecules induces the deviation of the linear correlation. Moreover, we have further derived the charge transfer mechanism, which leads to the preliminary determination of charge transfer. This work demonstrates the complicated underlying mechanism of charge transfer to enhance the understanding of the CE, which involves the coupling of various physiochemical effects. Depending on our preliminary explorations from the electronic view, further in-depth investigations from both experiments and theoretical calculations will benefit the optimization of TENG-based energy conversion and storage devices in a wide range of applications.

## Methods

### Calculation setup

DFT calculations implanted in CASTEP packages are performed to study the electron transfer of liquid–solid interface^45^. We choose the generalized gradient approximation (GGA) with Perdew-Burke-Ernzerhof (PBE) to describe the exchange-correlation energy^46–48^. Meanwhile, the cutoff energy of the plane-wave basis set was set to be 330 eV with ultrasoft pseudopotentials. We have applied the coarse quality for the k-points for the energy minimization based on the Broyden-Fletcher-Goldfarb-Shannon (BFGS) algorithm^49^. The convergence thresholds are set as 5 × 10^−5^ eV per atom for the total energy and 0.005 Å per atom for the inter-ionic displacement, respectively.

We have cleaved 4 × 4 supercells with five-layer thickness from the unit cell of different oxides as the solid surface in the liquid–solid surface. The introduction of water molecules is placed on the top of the solid surface. For the single-layered water, we choose the diamond, SiO_2_, TiO_2,_ and HfO_2_ with different dielectric functions. For comparison, we choose three different liquid systems including pure H_2_O, H_2_O+Na, and H_2_O+Na^+^. The additional consideration of Na^+^ aims to reproduce a similar microscopic environment as the proposed mechanism in experiments. For the multi-layered water, we have chosen oxides including ZnO, SnO_2_, MgO, HfO_2_, Ta_2_O_5_, and BaTiO_3_ with the same amount of water molecules for the unit areas. We only consider two systems: pure H_2_O and H_2_O+Na. The Na atoms are placed evenly between layers of water. In this work, all the water molecules are well ordered near the solid surface to reveals the change of electronic structures and interactions at the liquid–solid interface. Our previous works have confirmed that the amorphous arrangements of water molecules show similar coordination numbers and electronic structures with the crystalline water molecules with long-range order. We have guaranteed at least 15 Å vacuum space in the *z*-axis for geometry relaxations.

## Supplementary information

Supplementary Information

## Data Availability

The data supporting this study are available in the paper and Supplementary Information. All other relevant source data are available from the corresponding authors upon reasonable request.
