# Supplementary material for: Understanding contact electrification at liquid–solid interfaces from surface electronic structure
Source: Nat Commun. 2021 Mar 19;12:1752. doi: 10.1038/s41467-021-22005-6 (PMC7979908; doi:10.1038/s41467-021-22005-6)
Supplement: Supplementary file 1 — Supplementary Information [file 41467_2021_22005_MOESM1_ESM.pdf]

Supplementary Information

Understanding Contact Electrification at Liquid-Solid Interfaces from Surface Electronic Structure

Sun et al.

Supplementary Figures

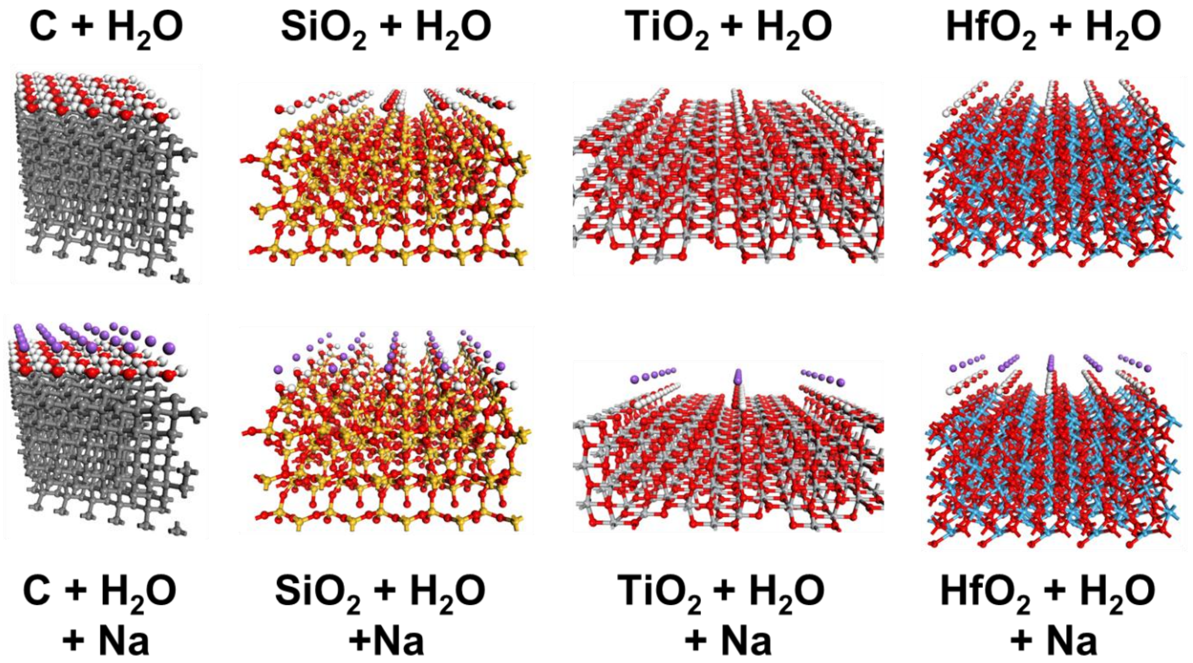

Supplementary Fig. 1 The simulation models for multi-layered water and solution in insulator diamond, dielectric insulator SiO_2, transition metal oxide TiO_2 and high-K oxide HfO_2.

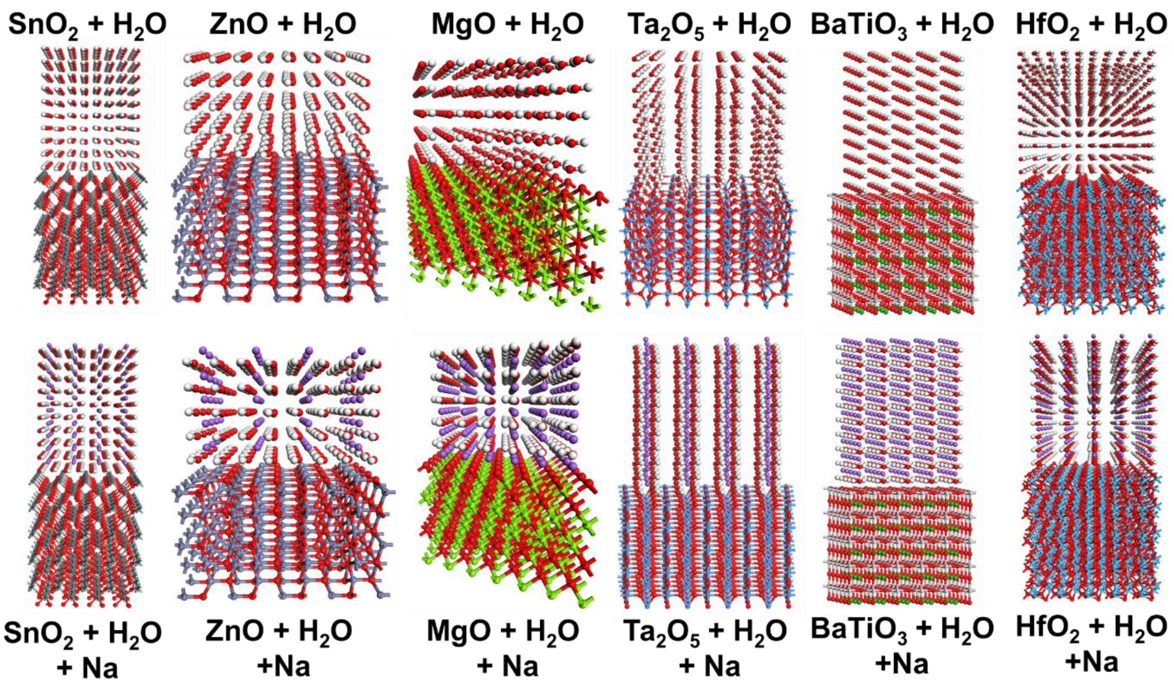

Supplementary Fig. 2 The simulation models for multi-layered water and solution in SnO_2, ZnO, MgO, Ta_2O_5 and HfO_2.

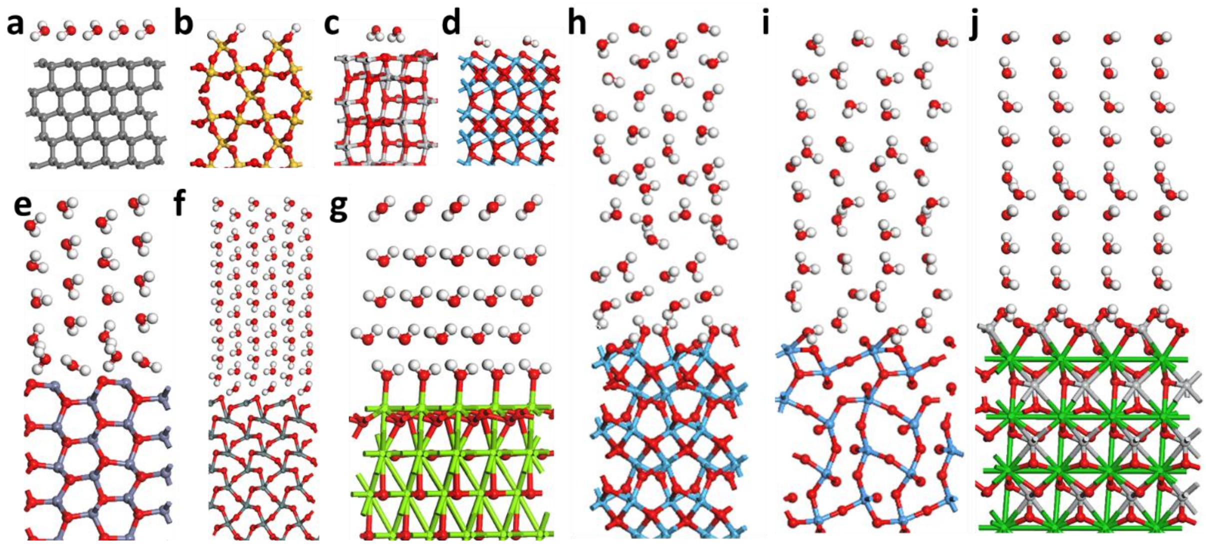

Supplementary Fig. 3 The side view of different solids after contacting with water molecules. Single-layered water on (a) Diamond; (b) SiO_2, (c) TiO_2, and (d) HfO_2. Multi-layered water on (e) ZnO; (f) SnO_2, (g) MgO, (h) HfO_2, (i) Ta_2O_5 and (j) $BaTiO_3$.

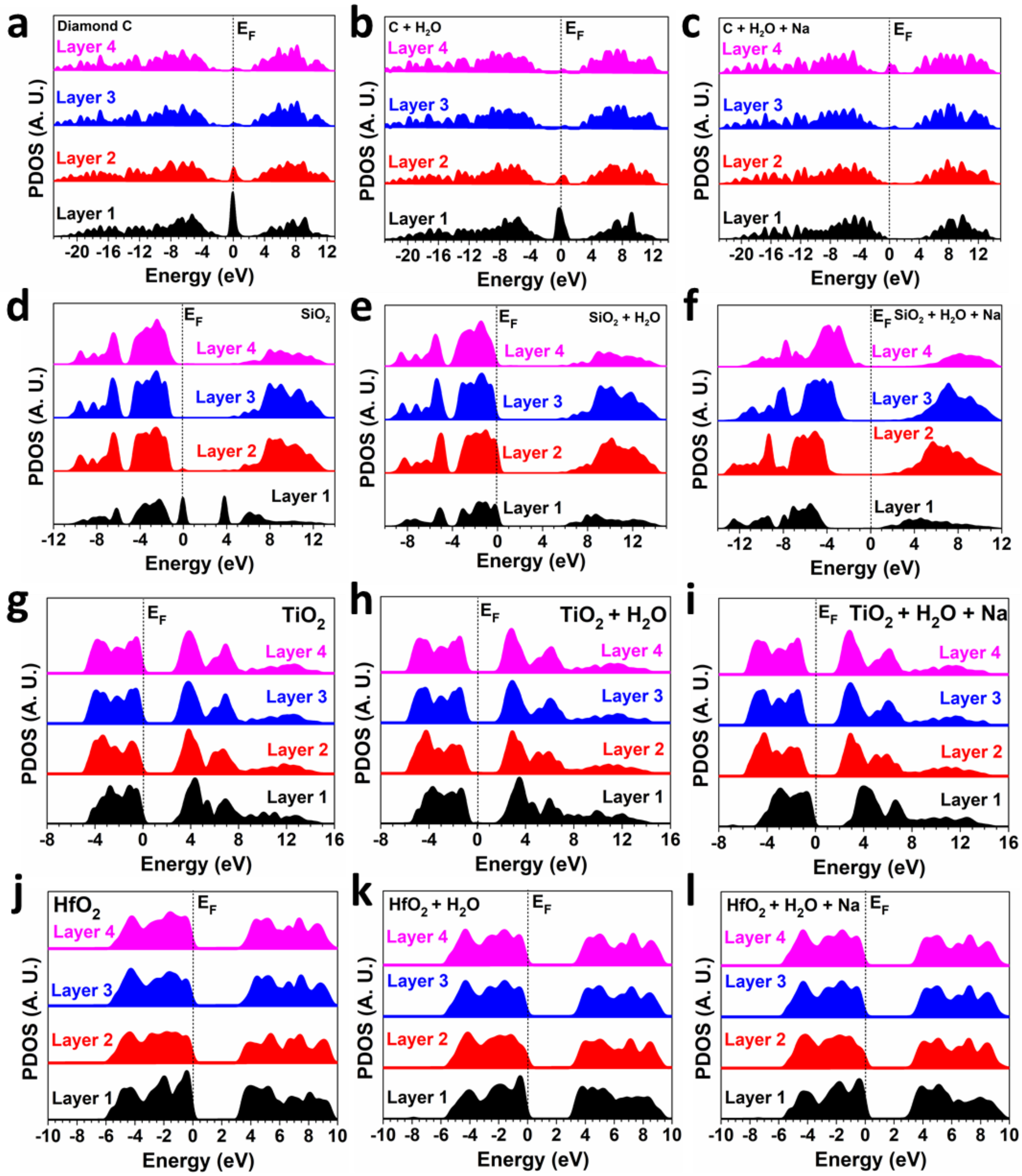

Supplementary Fig. 4 The layer-dependent TDOS of each solid after contacting with single-layered water. (a) diamond C, (b) C + H_2O and (c) C + H_2O + Na. (d) SiO_2, (e) SiO_2 + H_2O and (f) SiO_2 + H_2O + Na. (g) TiO_2, (h) TiO_2 + H_2O and (i) TiO_2 + H_2O + Na. (j) HfO_2, (k) HfO_2 + H_2O and (l) HfO_2 + H_2O + Na.

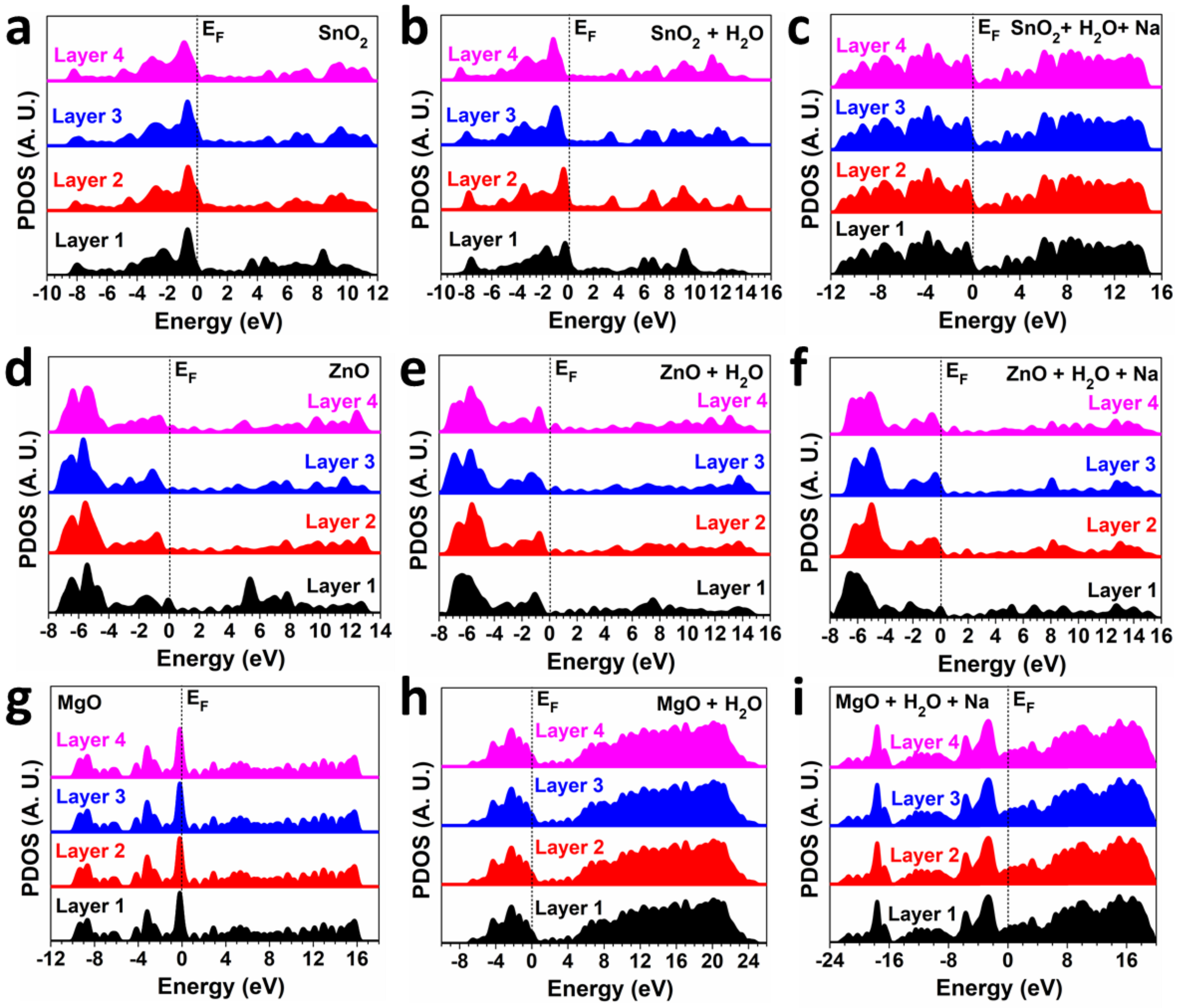

Supplementary Fig. 5 The layer-dependent TDOS of each solid after contacting with multi-layered water. (a) SnO_2, (b) SnO_2 + H_2O and (c) SnO_2 + H_2O + Na. (d) ZnO, (e) ZnO + H_2O and (f) ZnO + H_2O + Na. (g) MgO, (h) MgO + H_2O and (i) MgO + H_2O + Na.

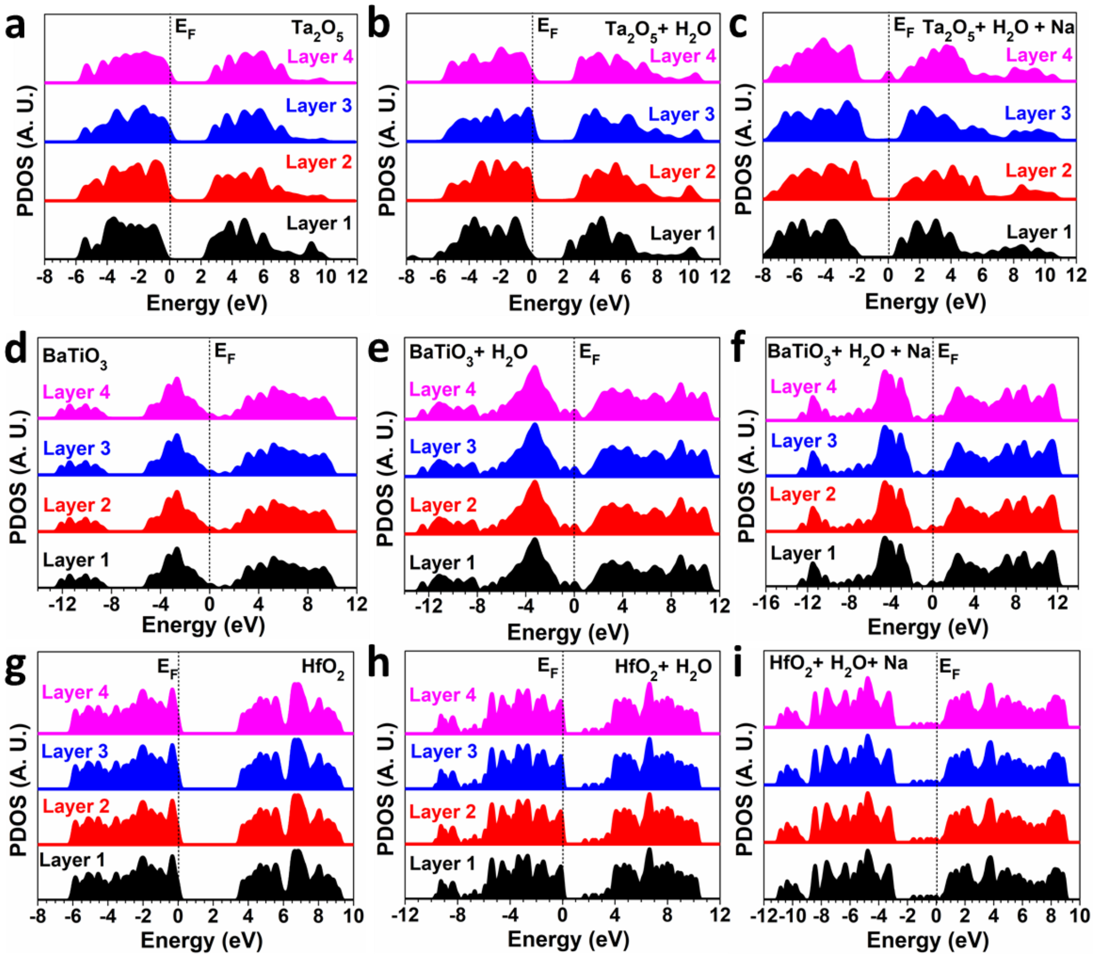

Supplementary Fig. 6 The layer-dependent TDOS of each solid after contacting with multi-layered water. (a) Ta_2O_5, (b) Ta_2O_5 + H_2O and (c) Ta_2O_5 + H_2O + Na. (d) $BaTiO_3$, (e) $BaTiO_3$ + H_2O and (f) $BaTiO_3$ + H_2O + Na. (g) HfO_2, (h) HfO_2 + H_2O and (i) HfO_2 + H_2O + Na.

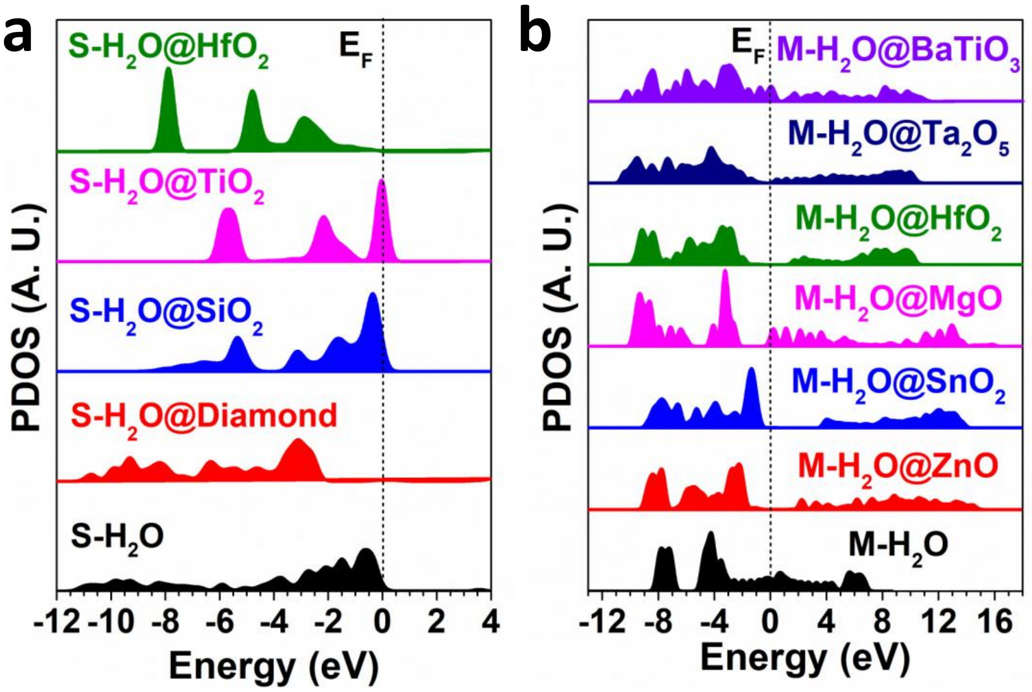

Supplementary Fig. 7 Electronic structure comparisons of water on different solid surfaces. (a) The single-layered water on different solid surfaces. (b) The multi-layered water on different solid surfaces.

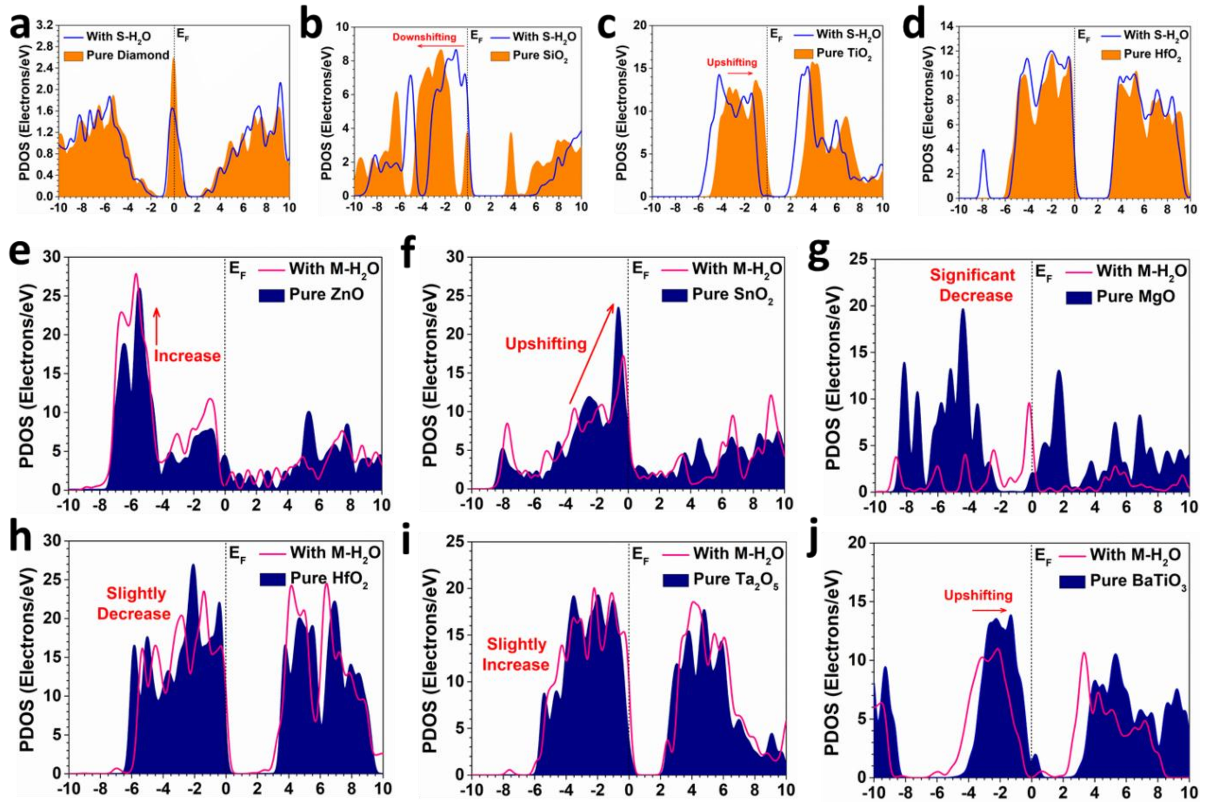

Supplementary Fig. 8 Electronic comparison of the top two-layers in the solids after contacting electrification with water. Single-layered water on (a) Diamond; (b) SiO_2, (c) TiO_2, and (d) HfO_2. Multi-layered water on (e) ZnO; (f) SnO_2, (g) MgO, (h) HfO_2, (i) Ta_2O_5 and (j) $BaTiO_3$.

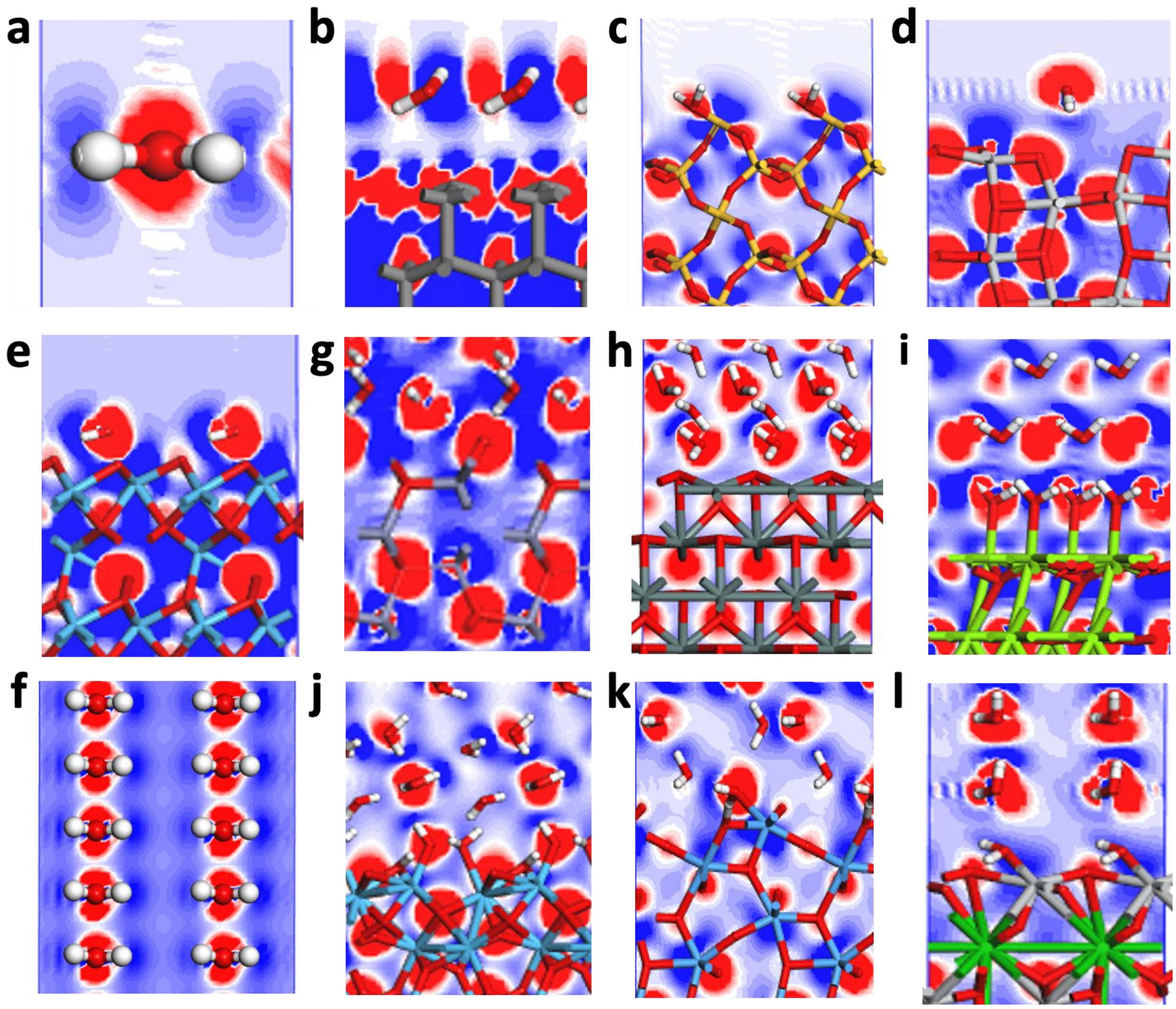

Supplementary Fig. 9 Electronic density difference of the water-solid interface after contact electrification with water. (a) Single-layered water. Single-layered water on (b) Diamond; (c) SiO_2, (d) TiO_2, and (e) HfO_2. (f) Multi-layered water. Multi-layered water on (g) ZnO; (h) SnO_2, (i) MgO, (j) HfO_2, (k) Ta_2O_5 and (l) $BaTiO_3$.

Supplementary Table

Supplementary Table S1. The summarized data for electronic parameters and charge transfer in different liquid/oxide systems

Solid	ΔE_{ads}(eV)	$\phi_{\text{Work Function}}$ (eV)	$\Delta\phi_{\text{Work Function}}$ (eV)	Charge Q (nC)	Dielectric Constant	P (pinning factor)
**SiO_2**	-2.48	6.46	0.185	1.44	2.49	0.82
**TiO_2**	0.02	6.11	-0.12	0.02	10.2	0.11
**HfO_2**	-1.52	5.33	-1.24	0.56	5.45	0.34
**ZnO**	3.10	5.03	-0.02	14.5	11.3	0.09
**Ta_2O_5**	-1.64	3.58	-2.76	0.06	4.87	0.40
**MgO**	11.11	4.83	0.25	9.23	6.77	0.23
**SnO_2**	13.54	6.16	-0.07	20.8	8.42	0.15
